# An in-depth Analysis of the Degree of Implementation of Integrated Care for Diabetes in Primary Health Care in Cambodia

**DOI:** 10.5334/ijic.7602

**Published:** 2024-12-04

**Authors:** Vannarath Te, Sereyraksmey Long, Wim Van Damme, Por Ir, Edwin Wouters, Josefien van Olmen

**Affiliations:** 1 School of Public Health, National Institute of Public Health, Phnom Penh, Cambodia; 2 Health Policy Unit, Department of Public Health, Institute of Tropical Medicine (Antwerp), Belgium; 3 Quality of Integrated Care, Department of Family Medicine and Population Health, the University of Antwerp, Belgium; 4 Health Policy Unit, Department of Public Health, Institute of Tropical Medicine (Antwerp), Belgium; 5 Management team, National Institute of Public Health, Phnom Penh, Cambodia; 6 Centre for Population, Family & Health, Department of Sociology, the University of Antwerp, Belgium; 7 Quality of Integrated Care, Department of Family Medicine and Population Health, the University of Antwerp, Belgium

**Keywords:** diabetes care, integrated care, innovative care for chronic conditions, primary health care

## Abstract

**Introduction:**

With the rising prevalence of type 2 diabetes (T2D), three care initiatives for T2D are being scaled-up in Cambodia to improve availability and accessibility of integrated care for T2D: (1) *hospital-based care*, (2) *health centre-based care*, and (3) *community-based care*. This case study aims to share learnings from an in-depth analysis of the level of integrated care implementation in these care initiatives for T2D in Cambodia.

**Description:**

Twenty public health facilities in five operational districts were assessed on six integrated care components: (1) early detection and diagnosis, (2) treatment in primary care services, (3) health education, (4) self-management support, (5) structured collaboration, and (6) organisation of care. Two raters independently scored each facility on a 0–5 scale based on multiple sources of data and reached a consensus.

**Discussion:**

The in-depth analysis showed that the three care initiatives were not implemented in an integrated manner, with low implementation scores (1 or 2 out of 5) in all selected settings. The presence of *health centre-based care* was associated with higher scores for early detection and diagnosis and treatment in primary care services, while the presence of *community-based care* was related to structured collaboration and organisation of care.

**Conclusion:**

The evidence suggests that while each care initiative has its potential contributions towards integrated care for T2D, the three care initiatives should be effectively implemented in an integrated manner in order to potentially produce the desired outcomes.

## Introduction

In 2021, globally, 537 million adults aged 20–79 years were living with type 2 diabetes (T2D), of which over three quarters were living in low- and middle-income countries (LMICs) [1]. While the increase in T2D prevalence has been the most rapid in LMICs, the capacity of primary health care (PHC) for screening, diagnosis, treatment, and care management is still limited in these contexts [2]. Cambodia, a LMIC in the World Health Organisation (WHO) Western Pacific region – the region with the highest number of adults living with T2D [1] – has experienced a significant increase in T2D prevalence in the last 10 years: 9.6% of adults aged 18–69 were found to live with T2D in 2016, while it was 2.9% amongst the 25–64 age group in 2010 [3]. The Cambodian PHC system has limited capacity for meeting the needs of the population at risk and those already living with T2D: more than two-thirds of the population have not had their blood glucose level tested; more than half of those living with T2D are not receiving treatment; and only few of those receiving treatment achieve recommended treatment targets [3].

T2D that is not properly and adequately managed can lead to complications [4] – the major complications are cardiovascular diseases [2,5] which accounted for 24% of the Cambodia’s total deaths in 2018 [6]. Lack of access to T2D care including anti-diabetic medicines has been found to negatively affect outcomes [7]. Due to its asymptomatic and progressive nature, incurability, and chronicity, T2D management requires a continuum of preventive, curative and care services in place, preferably in an integrated manner, to prevent or delay complications [8,9]. Integrated care implies that involved care providers share necessary information and coordinate resources in an effective and efficient manner across the care continuum and involved care providers [10]. The WHO developed the Innovative Care for Chronic Conditions (ICCC) framework for health system transformation towards the integrated care for chronic disease management [8]. The ICCC framework was adapted from the Chronic Care Model [11] which has been found to be effective for the management of T2D in primary care in terms of improved clinical outcomes [12,13,14,15,16,17,18,19]. The ICCC framework, however, is more comprehensive and applicable to a wider international context including LMICs [8,20]. At the implementation level, to achieve better outcomes for chronic conditions, the ICCC framework gives emphasis on a triad interaction between people with chronic conditions and their families, the health care team, and community partners; with support from both the health care organisation and the community. An assumption is that presence of the health care component and the community component will contribute to better implementation of integrated care. In developing health care systems like Cambodia, the system is still building the different components for non-communicable disease (NCD) care through various care initiatives. It is unclear how the combination of different care initiatives contributed to integrated care. Therefore, this case study aims to share learnings from an in-depth implementation analysis of the different combination of care initiatives for T2D in Cambodia, according to the ICCC framework.

## Ethical Approval

The case study obtained ethical clearance from the National Ethics Committee for Health Research in Cambodia with reference number 105 NECHR and the Institutional Review Board of Institute of Tropical Medicine (Antwerp) with reference number 1323/19.

## T2D Care Practice in Cambodia

In Cambodia, the Ministry of Health oversees the overall health system, which is pluralistic, consisting of both public and private providers (including non-profit organisations). Cambodia follows the PHC approach to operating public health care on a district health system model. In this approach, one operational health district (OD) contains approximately 10–25 health centres (HCs) providing primary care – commonly known as a minimum package of activities [21] – to communities with support of community health workers and a referral hospital (RH) which provides secondary care complementary to the HCs. Severe cases can be further referred for tertiary care at a national hospital [21]. Despite the different levels of care, there is no strict practice of using the primary care provider as a gatekeeper. In 2022; across the 25 provinces and capital in the country, there were 103 ODs, 120 referral hospitals, and 1,269 health centers [22].

Three care initiatives for T2D are being scaled-up across the 103 ODs to improve the availability and accessibility of the integrated care for T2D in Cambodia: (1) *hospital-based care*, (2) *health centre-based care*, and (3) *community-based care*. The care initiatives are mentioned in the 2019 national standard operating procedure for the management of T2D and hypertension in primary care [23]. This standard operating procedure was adapted from the WHO package of essential NCD interventions (WHO PEN) [24] with the intention to apply PHC approach to T2D care in which HCs offer the continuity of care and coordination across the care levels in the OD – HCs implementing the standard operating procedure are defined as HCs with WHO PEN. The WHO PEN was piloted in four HCs in 2015. In 2023, there were 252 HCs (out of 1,269) implementing the WHO PEN in the country [25]. *Hospital-based care* is provided at RHs focusing on confirmation of diagnosis, treatment initiation and treatment of serious or complicated T2D cases. *Health centre-based care* is provided at the HCs with WHO PEN to screen for T2D (targeting the population aged 40 and over), provide follow-up care for mild and stable T2D cases without complications, and offer counselling on positive lifestyle changes. *Community-based care* is offered by community health workers – operating in either 1) a village health support group formally recognised by the Ministry of Health or 2) a peer educator network supported by a Cambodian non-governmental organisation called MoPoTsyo – providing support to the HCs. The peer educators – also people living with T2D – offer self-management support to people with T2D in their network and assist them to have access to physician consultation, laboratory tests, and low-cost medicines through a revolving drug fund program [26]. The village health support group, on the other hand, perform multiple functions in the community including health awareness raising. They are usually members of commune councils. Their function in the managment of T2D and hypertension is limited. Therefore, the *community-based care* for T2D in this case study only includes the one provided by the peer educator network of MoPoTsyo.

Through stakeholder interviews with program managers of the three care initiatives at the national level, we purposively selected ODs based on availability of the care initiatives to ensure a maximal spread, which embodied the full spectrum of combination of care initiatives in Cambodia: 1) the co-existence of the three care initiatives (*hospital-based, health centre-based, and community-based*), 2) *hospital-based care only*, 3) *health centre-based care only*, and 4) *community-based care only*. It is noted that based on the implementation arrangement, HCs can implement WHO PEN only if there is the availability of an NCD clinic at the RH [23]. Therefore, the *health centre-based care* which is provided by the WHO PEN health centre is supported by the NCD clinic. We further selected ODs with low and high coverage of the WHO PEN health centres to see the influence level of WHO PEN; as a result, five ODs in different provinces were selected in this study.

**Figure 1:**
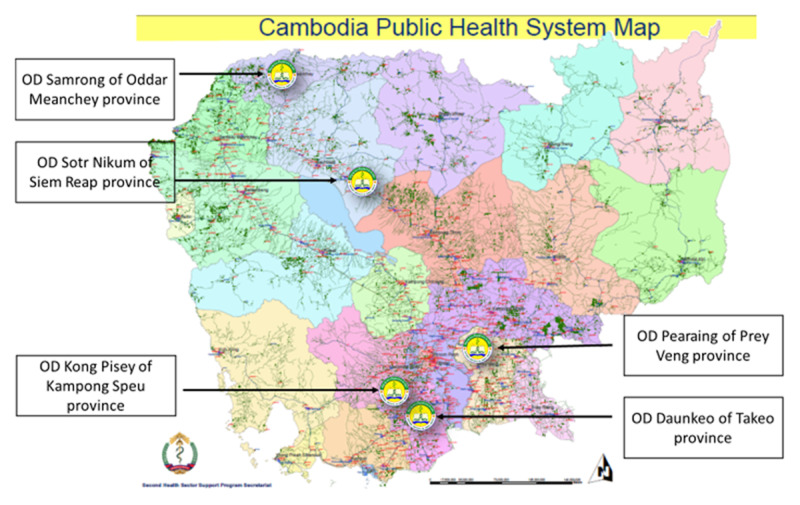
Locations of the five selected ODs in Cambodia.

The selected five ODs included: 1) OD Daunkeo in Takeo province comprised the three care initiatives together: The *hospital-based care* at the Chronic Disease Clinic [27] providing treatment and care to people with T2D and/or hypertension; the *health centre-based care* in eight out of 15 HCs; and the *community-based care* provided by the MoPoTsyo peer educator network. 2) OD Pearaing in Prey Veng province in which only *health centre-based care* was available through eight out of nine HCs (i.e. high coverage). 3) OD Sotr Nikum in Siem Reap province in which *health centre-based care* was available through five out of 25 HCs (i.e. low coverage) and hospital-based care through the Chronic Disease Clinic in the Sotr Nikum RH. This OD also has a historical and significant influence from various development partners and non-governmental organisations (a contextual factor). 4) OD Kong Pisey in Kampong Speu province comprised only the *community-based care* model organised by the peer educator network, although the peer educator network made the arrangement with the RH to provide physician consultations for people with T2D in the network once a week. 5) OD Samrong in Oddar Meanchey province conducted the *hospital-based care* in the NCD clinic of the Oddar Meanchey RH – the only public provider of T2D care in the OD. Figure 1 shows locations of the study settings in Cambodia. Table 1 shows the five ODs hosting the three care initiatives either individually or in co-existence at the time of data collection from June-August 2019.

**Table 1:** Five ODs and the availability of care initiatives for T2D.


OD	PROVINCE	COMBINATION OF CARE INITIATIVES	EXISTING CARE PROVISION

1) Daunkeo	Takeo	Co-existence of the three care initiatives	NCD clinic + WHO PEN + Peer Educator Network

2) Pearaing	Prey Veng	Health centre-based care	NCD clinic + WHO PEN (high coverage)

3) Sotr Nikum	Siem Reap	Health centre-based care with context	NCD clinic + WHO PEN (low coverage)

4) Kong Pisey	Kampong Speu	Community-based care	Peer Educator Network

5) Samrong	Oddar Meanchey	Hospital-based care	NCD clinic


In each OD, we assessed the RH and three randomly selected HCs – each OD is a unit of analysis. In total, five RHs and 15 HCs were assessed. Relevant management team and staff members in the selected health facilities were interviewed as a part of data collection. The ICP grid which stands for Integrated Care Package Implementation Assessment Framework was the instrument we used to assess the integrated care for T2D and was developed based on two assessment tools that have been validated and widely used in high- and low-income settings to assess integrated care for chronic conditions: 1) the Assessment of Chronic Illness Care Form [28] and the ICCC Framework Situation Assessment Form [29]. The ICP grid allowed us to measure six components of the integrated care including: (1) early detection and diagnosis, (2) treatment in primary care services, (3) health education, (4) self-management support, (5) structured collaboration, and (6) organisation of care. Table 2 shows the questions or items in the grid. The ICP grid has already been successfully applied in Slovenia and Belgium [30,31]. It was translated into Khmer (Cambodian national language) and field tested before the actual data collection in the five ODs. Appendix 1 shows details of the ICP grid.

**Table 2:** Questions of each ICP component in the grid.


ICP COMPONENTS	QUESTIONS/ITEMS

**Component 1:** Facility-based identification of patients with T2D	1.1. To what extent, is screening for T2D performed among patients at a visit? 1.2. To what extent, are equipment and materials necessary for diagnosing patients for T2D available at the facility? 1.3. To what extent, are health care staff or service providers competent to perform diagnosis for T2D at the facility? 1.4. To what extent, is the follow-up of the patients after the screening, testing and diagnosis of T2D organised?

**Component 2:** Treatment of T2D by primary care providers using standardised protocols	2.1. To what extent, are written guidelines of care and treatment accessible to primary care providers for T2D? 2.2. To what extent, are primary care providers in charge competent to provide treatment for patients with T2D? 2.3. To what extent, are the essential medications for T2D available in the primary care setting? 2.4. To what extent, do primary care providers have necessary laboratory access? 2.5. To what extent, have primary care providers received training for treating T2D? 2.6. How comprehensive is treatment beyond medication prescription for T2D (including measuring of body mass index, waist circumference, blood pressure, cholesterol level, renal function, screening for complications – foot examination, eye problem, macrovascular disease, depression)? 2.7. To what extent, are medication reviews undertaken in the elderly with T2D in order to avoid polypharmacy, hypoglycemia and renal dysfunction?

**Component 3:** Health education and counselling to patients with T2D by non-physician care providers	3.1. To what extent, do patients with T2D receive information on how to reduce health risks by non-physicians? 3.2. To what extent, are patients informed about the chronic condition of T2D by non-physicians (including the expected course, expected complications, and effective strategies to prevent complications and manage symptoms)? 3.3. To what extent, are non-physicians trained to provide health education and counselling to patients with T2D? 3.4. To what extent, are health education or counselling materials accessible to non-physicians for T2D?

**Component 4:** Self-management support to patients and their informed caregivers with tools for adherence and monitoring	4.1. To what extent, are patients offered self-management training for T2D (for example, to improve adherence to medications, proper nutrition, having self-monitoring tools at home, consistent exercise, tobacco cessation, and maintain other healthy behaviours)? 4.2. To what extent, do health care staff or community health workers support patients’ self-management efforts on a continuous basis for T2D? 4.3. To what extent, are health care staff or community health workers competent to perform self-management training? 4.4. To what extent, does the patient have access to materials for self-monitoring for T2D, for instance, glucose meter/glucose test strips? 4.5. To what extent, are informal caregivers/non-medical involved in the self-management process for T2D? 4.6. Are the concerns of patients and families addressed? 4.7. Are patient treatment plans agreed with patients, reviewed and written down?

**Component 5:** Structured collaboration between health care workers, community actors, and patients and caregivers	5.1. To what extent, is there an identified “care coordinator” who serves as the overseer and director of a patient’s care, ensuring that efforts of all involved health care workers, community actors, and patients and caregivers are integrated and coordinated for T2D? 5.2. To what extent, do the health care organisation and the community have complementary functions, that is, the community organisation fills gaps in services that are not provided in formal health care for T2D? 5.3. To what extent, are referral practices systematically organised for T2D? 5.4. To what extent, does cooperation between health care workers and other professionals and community actors occur for T2D? 5.5. To what extent, is the traditional hierarchy flattened and moved away from physician-dominated models for T2D?

**Component 6:** Organisation of care, delivery system design and clinical information systems	6.1. To what extent, are ongoing quality improvement routine activities among health care workers organised? 6.2. To what extent, do information systems gather and organise data about epidemiology, treatment, and health care outcomes? 6.3. To what extent, do information systems serve as a reminder function for patient specific prevention and follow-up services (e.g. to identify patients’ needs, follow-up and plan care, monitor responses to treatment, and assess health outcomes)? 6.4. To what extent, is feedback about the performance provided to the team and its members? 6.5. To what extent, is an appointment system with planned visits used?


Each question of the ICP grid was given a score based on the synthesis of multiple sources of data collection. The data sources were obtained from: 1) participant observation at the health facilities during the operations; 2) key informant interviews with the director or deputy director of the respective provincial health department, OD, and RH; 3) focus group discussions with health care staff from the respective NCD clinic and HCs and with community health workers including the village health support group and peer educator network; 4) focus group discussions or in-depth interviews with patients with T2D who were either referred or selected at the health facilities; and 5) inspection of documents at the health facilities (e.g. management book, patient registry book, outpatient record book, patient files, etc.). In total, thirteen focus group discussions were conducted (four to six people per group) and 16 key informant interviews were carried out. Two raters independently scored each health facility and then reached a consensus final score through discussion. The scoring system was based on a 0–5 scale: 0 = ‘no implementation’, 1 = ‘little implementation’, 2 = ‘lower moderate implementation’, 3 = ‘upper moderate implementation’, 4 = ‘almost complete implementation’, and 5 = ‘full implementation’. Detailed explanation of each scale for each question can be found in Appendix 1.

For each OD, a score was generated per ICP component based on the mean of the scores for all the questions under each component. The score for each question, nevertheless, was based on the mode of the assessed health facilities, given that the data were categorical by nature. Potential contributions were identified when presence of a particular care initiative consistently increased a particular component score across the selected ODs.

## Discussion

**Figure 2:**
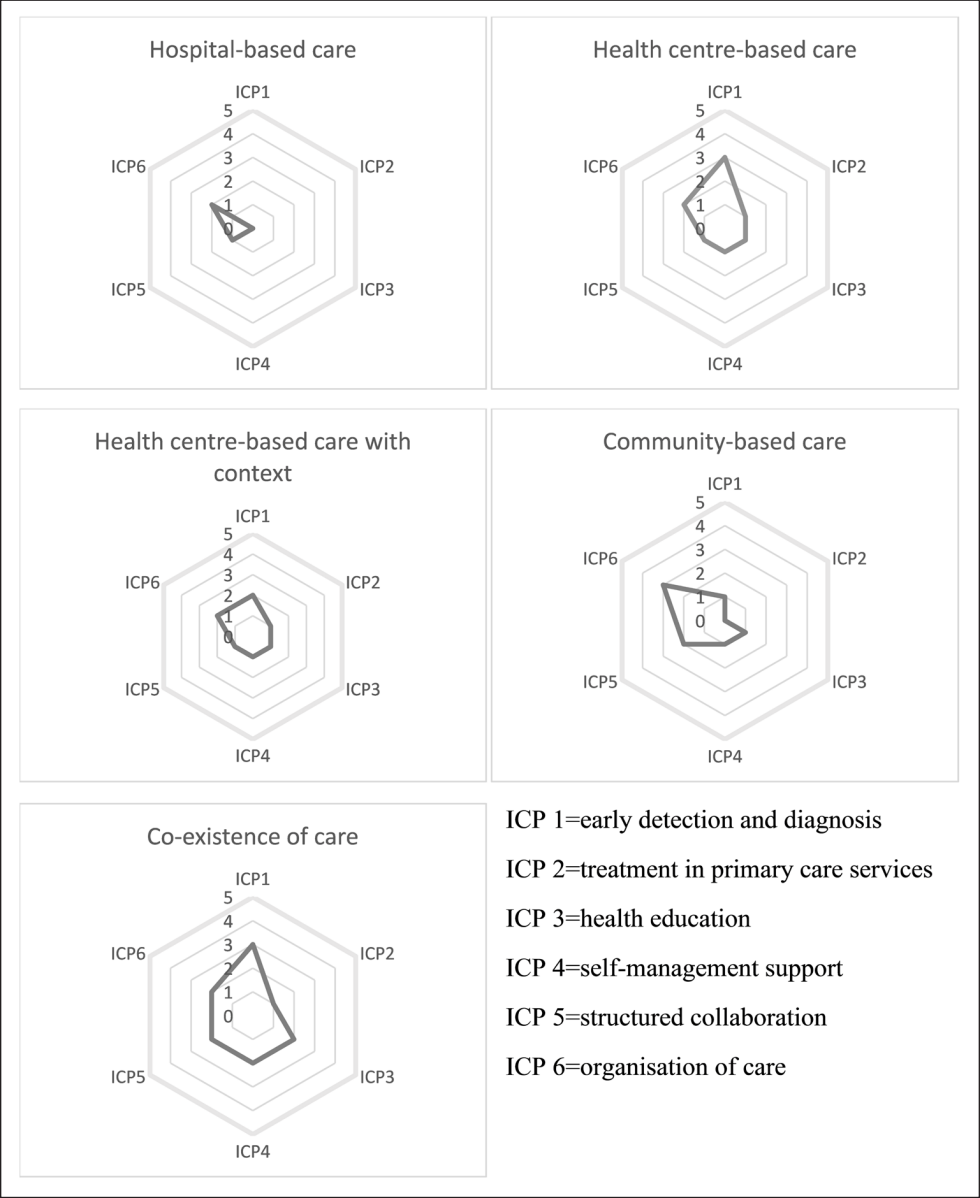
Spider chart representing the ICP components of the care initiatives either individually or in co-existence.

Generally, the implementation scores were low across the ICP components in all the selected ODs—around 2 out of 5 which is ‘lower moderate implementation’. The presence of *health centre-based care* tended to produce higher scores for early detection and diagnosis (ICP1) and contributed minimally to treatment in primary care services (ICP2), while the presence of the *community-based care* for structured collaboration (ICP5) and organisation of care (ICP6). The co-existence of care was likely to generate better scores across the ICP components. Figure 2 shows the spider chart representing the ICP components of the care initiatives either individually or in co-existence.

With the *health centre-based care*, HC staff received training on T2D at the start of implementation, and HCs were supplied with equipment and materials for screening and health education. However, program sustainability and implementation fidelity were questionable. When the equipment and materials were out of order or stock, related activities were usually halted. There was evidence of expired testing strips and of unused equipment and materials that were provided, indicating a low priority given to the program.

The *health centre-based care* emphasized and reinforced the screening activity for T2D at HCs, especially in the population aged 40 and over, but diagnosis and initial treatment still had to be made at the RH. T2D treatment at the HCs was still restricted [23] as HCs were mostly operated by nurses and midwives who were not adequately informed about T2D treatment in their formal education. Hence, the Central Medical Store, a governmental institution responsible for storing and dispensing medical equipment and drugs, did not supply anti diabetic medicines to HCs. Although the national standard operating procedure allows the HCs to refill prescriptions for stable cases of T2D [23], unavailability and inadequacy of antidiabetic medicines at the HCs were widely evidenced. This issue of inadequacy in essential medicines such as Metformin, Gliclazide, etc. was also highlighted in a study in Myanmar [32]. The shortage of medicines, nevertheless, was not the case in a pilot study in Bhutan where strong supportive supervision from the higher up was available [33].

Peer educator networks in ODs with the *community-based care* were likely to increase scores for structured collaboration (ICP5). However, the network was not formalised in the public health care system and mainly received support from the non-governmental organisation. The linkage function of the peer educator is therefore vulnerable to change. This is seen in the OD with co-existence of care, where the peer educator network had been handed over to the local health governance and technical or financial support from the organisation disappeared, rendering the network dysfunctional. From the community side, the peer educators have the potential to play a complementary role filling in the gaps in T2D service provision, as also evidenced in other studies [26,34]. A considerable number of systematic reviews in other countries have found that peers can effectively provide self-management support to patients as they share similar experience, knowledge, and other characteristics to the patients [35,36]. Likewise, lay people (such as the village health support group) who are not necessarily patients themselves can also provide effective self-management support to patients [37,38,39,40,41]. Community health workers, either peer educators or village health support group, need to be equipped with necessary software (knowledge and skills) and hardware (medical equipment and supplies), and motivated to provide complementary care to that of the health care workers in order to support government plans to strengthen the PHC [8,42]. Regarding the organisation of care (ICP6), peer educator network’s database was observed in the OD with the *community-based care* to be a crucial local health information system for the continuity of care.

The co-existence of care seemed to have better scores due to the combined contributions, but the co-existence did not automatically generate synergism necessary for the optimum integrated care for T2D. The three care initiatives were not implemented in an integrated way but rather in isolation with limited interaction between them. Working mechanisms facilitating the integrated care for T2D in terms of shared necessary information and coordinated resources [10] were not observed. There was no proper system for following-up patients for the continuity of care – the peer educator network in the OD with co-existence of care was not functioning optimally. The referral system between the communities, HCs, and RHs was dysfunctional. The patient record was still paper-based and the form was usually filled with insufficient information. At the NCD clinic of the RH, there was use of a database, but there was ineffective and inefficient use of it in connection to other public health facilities at different levels of care. The peer educators did not work closely with the village health support group or even the HCs. With insufficient resources, capacity and commitment, management teams at the OD and the provincial health department levels could not provide regular supervision and support for the implementation.

## Lessons Learnt

At the practice level:

- The co-existence of care initiatives does not automatically generate the synergism necessary for the optimum integrated care for T2D. Working mechanisms facilitating the integrated care for T2D are needed. The mechanisms could include: 1) improving leadership and management skills among health care workers and community leaders, 2) improving digital literacy among health care workers and community health workers for case management, and 3) promoting a multidisciplinary, inclusive, collaborative, and complementary team in which healthcare workers with special training in T2D (such as diabetes educators) take the lead.
- Health promotion and prevention activities are crucial for the continuum of care for T2D and should be equally emphasised. Raising awareness of the T2D burden through health education in the community should be prioritised.

At the policy level:

- Implementing integrated care for T2D through existing models is possible if there is enabling policy to provide the implementation context in all models and for the care actors. HCs should be allowed to make a diagnosis and initiate treatment for non-complicated T2D cases. As most HCs in Cambodia are operated by nurses, pre-service education for nurses should be upgraded so that graduates have necessary competencies in making a diagnosis and initiating treatment for non-complicated T2D cases. Anti diabetic medicines should be put on the essential drug list for primary care level. All relevant community health workers including peer educators should be formalised with financial support.
- Health care facilities need a stable supply of resources in order to generate trust among the patients. Decentralisation for local health governance needs to be strengthened, and the social health protection scheme for T2D-related services needs to be expanded.

## Conclusion

This case study shares lessons on how we can use the ICP grid to systematically analyse the degree of implementation of the integrated care for T2D in the Cambodian PHC. Each of the three care initiatives has its potential contributions towards different care components of the ICP. The *health centre-based care* was likely to produce higher capacity in early detection and diagnosis (ICP1) and treatment in primary care services (ICP2), while the *community-based care* tended to produce better results for structured collaboration (ICP5) and organisation of care (ICP6). The close examination of co-existence of care showed that they were not implemented in an integrated way and as intended in the written guidelines. For policy implications, while scaling-up the three care initiatives, further effort should be put to explore factors enabling the three care initiatives to be implemented in an integrated way according to the ICCC framework.

## Additional File

The additional file for this article can be found as follows:

10.5334/ijic.7602.s1 Appendix 1. Integrated Care Package Implementation Assessment Framework _ ICP Grid.
