# Supplementary material for: An in-depth Analysis of the Degree of Implementation of Integrated Care for Diabetes in Primary Health Care in Cambodia
Source: Int J Integr Care. 2024 Dec 4;24(4):11. doi: 10.5334/ijic.7602 (PMC11623098; doi:10.5334/ijic.7602)
Supplement: Appendix 1. — Integrated Care Package Implementation Assessment Framework _ ICP Grid. [file ijic-24-4-7602-s1.pdf]

Integrated Care Package (ICP) Implementation Assessment Framework _ ICP Grid

(This grid has been modified from the original work to fit the topic of type 2 diabetes care in Cambodia.)

General explanation of the ICP grid:
Most of the questions in this assessment framework are adapted from the Innovative Care for Chronic Conditions (ICCC) Framework situation assessment. We aim to rate the integrated care package at a particular Unit of Analysis (an Operational District including a referral hospital and a number of health centers). The grid includes questions on the five components of the ICP and additional questions on overarching axis that relate to quality control (component 6). The ICP components measure both structure (are the necessary structures present?) and process (are processes done consistently and with which level of depth?).

Methods for filling the grid:
The ICP grid is not a direct data collection tool. It is rather a meta-analysis or synthesis of multiple data sources. The grid should be filled during analyses of data collected from the multiple sources (for the triangulation purpose). Two raters are required to fill the grid independently. A consensus between the raters should be reached after checking the collected data. Data are collected through:

- Key information interviews with representatives (director or deputy director) from the respective provincial health department, operational district, and referral hospital
- Focus group discussions with health care staff from health centers and the NCD clinic of the referral hospital
- Focus group discussions with community health workers including village health support group and peer educator network
- Focus group discussions or in-depth interviews with people living T2D
- Direct observations of the health facility infrastructure, organisation of work, patient flow, interactions of patients with health care workers
- Inspection of records or documents at the health facilities: management books, patient registries, and randomly selected patient files

Grading and scoring:
Response to each question is graded using a scale of 0 to 5 with an explanation of each grade.

- 0 : No implementation of ICP
- 1 : Little implementation of ICP
- 2-3 : Moderate implementation of ICP
- 4 : Almost complete implementation of ICP
- 5 : Full implementation of ICP

Note on terminology:

- BMI : Body mass index
- BP : Blood pressure
- HCW : Health care workers including both physicians and non-physicians
- Physicians : medical doctors
- Non-physicians : nurses or community health workers or other HCWs
- NCD : Non-communicable disease
- T2D : Type 2 Diabetes

Additional text to instruction for researchers filling the grid, especially on how to grade COMPETENCE:
They need to be well aware of the national guidelines (or golden standard where it is available) on how to diagnose, how to treat, how to provide information and self-management support. They will need to rate the competence of health care providers by observing providers and sometimes talking with providers, to see whether they act in according with the guidelines. This is an important aspect of field training.

Operational District: _______________   Province: ________________

Assessment Date: __________________

Analyzed and scored by: ____

**ICP Component**	**Response**						**Verified by**	**Justification**
**Component 1:**<br>**Facility based identification of patients with T2D**<br>*Facility: place (health center, hospital, clinic, home, headquarter) where care is provided*	**No or little implementation**		**Moderate implementation**		**Almost complete or full implementation**			
1.1. To what extent, is screening for T2D performed among patients at a visit? [PROCESS]	**0**<br><br>Not at all	**1**<br><br>Only on patient's initiative, not based upon HCW thinking of it (client-based)	**2**<br><br>When diabetes symptoms are present or required by other conditions	**3**<br><br>When required by risk factors but not consistently done	**4**<br><br>Consistently done in a group of patients defined by risk factors (almost everyone).	**5**<br><br>Everyone who needs to be tested gets tested.	-Asking health care staff (supported by observation and checking records at the triage)	
1.2. To what extent, are **equipment and materials** necessary for diagnosing patients for **T2D** available at the facility? [STRUCTURE]	0<br><br>Not at all	1<br><br>Available but not functional	2<br><br>Partially equipped – some parts are not functional	3<br><br>Equipped for a small number of patients	4<br><br>Fully equipped for almost everyone	5<br><br>Fully equipped for everyone	-Asking health care staff (supported by observation at the facility)	

1.3. To what extent, are health care staff or service providers **competent** to perform **diagnosis for T2D** at the facility? [STRUCTURE/EDUCATION]	0<br><br>Not at all	1<br><br>Know but cannot perform properly	2<br><br>Perform with guidance from others	3<br><br>Properly perform but cannot interpret the results	4<br><br>Properly perform with limited interpretation of the results	5<br><br>Properly perform with clear interpretation of the results	-Asking health care staff if they are educated about diagnosis criteria for T2D	
1.4. To what extent is the **follow-up of the patients after the screening, testing and diagnosis of T2D** organised? [PROCESS]	0<br><br>No follow up	1<br><br>Patients are referred for diagnosis/ therapy but no follow up	2<br><br>Follow up only on patient's initiative	3<br><br>Follow up on if positive diagnosed and not follow up if negative with high risk	4<br><br>Follow up for both positive and negative with high risk	5<br><br>Care is organised and planned for every patient and they are called if non-attending	-Asking the health care staff	
**Component 2**: **Treatment of T2D by primary care providers using standardized protocols**<br>*Primary care providers: first line of care providers (not including those at hospitals)*	**No or little implementation**		**Moderate implementation**		**Almost complete or full implementation**			
2.1. To what extent, are **written guidelines of care and treatment** accessible to primary care providers for T2D? [STRUCTURE]	0<br><br>Not available at all	1<br><br>Some guidelines are available but not used in daily practice or difficult to access	2<br><br>Easy accessible guidelines but not recently updated and not encouraged	3<br><br>Easy accessible guidelines and recently updated or their use is encouraged	4<br><br>Recent updated guidelines are available and their use is encouraged through posters and other educational process etc.	5<br><br>Recent updated guidelines are available and integrated in daily practice through reminders (pop-ups) in electronic medical record tailored to each patient	-Asking the providers to show guidelines: how easy/difficult the health care staff can show the guidelines when asked to present them.<br>-Observation at the facility	

2.2. To what extent, are primary care providers in charge **competent to provide treatmen**t for patients with T2D? [STRUCTURE/EDUCATION]	0<br><br>No knowledge at all	1<br><br>Have some non-pharmacolog ical knowledge	2<br><br>Have non-pharmacological knowledge and skills	3<br><br>Have detailed knowledge and non-pharmacological skills plus basic pharmacological knowledge	4<br><br>Have all detailed knowledge about pharmacological treatment	5<br><br>Have detailed knowledge and know how to treat complications	-Asking the health care staff	
2.3. To what extent are the **essential medications** for T2D available in the primary care setting? [STRUCTURE]	0<br><br>Not at all	1<br><br>Only some medications available but stock out	2<br><br>Accessible to one type of medicines	3<br><br>Accessible to two or more of medicines	4<br><br>Accessible to two or more of medicines and indicated	5<br><br>Fully accessible to all the essential medicines (including insulin) and indicated	-Asking the health care staff<br>-Checking the drug store against anti-diabetic medicines listed in the national guidelines)	
2.4. To what extent, do primary care providers have necessary **laboratory access**? [STRUCTURE]	0<br><br>Not at all	1<br><br>All testing items referred to the referral hospital	2<br><br>Limited testing items by rapid tests	3<br><br>All testing items by rapid tests	4<br><br>Limited testing items by own laboratory	5<br><br>All testing items by own laboratory	-Asking the health care staff<br>-Checking the laboratory capacity at the facility	
2.5. To what extent have primary care providers received **training for treating** T2D? [STRUCTURE/EDUCATION]	0<br><br>Not at all	1<br><br>On the job training or when the service started	2<br><br>Part of the formal education to obtain certificate needed to do the job	3<br><br>as in 2 plus sporadic extra trainings on the topic	4<br><br>As in 2 plus systematically extra trainings on the topic	5<br><br>As in 2 plus systematically extra obligatory trainings on the topic, with innovative methods	-Asking the health care staff<br>-Checking details of trainings (when? trained by whom? how long? about what?)	

2.6. How comprehensive is **treatment beyond medication prescription for T2D** (including measuring of BMI, waist circumference, BP measurements, cholesterol levels, renal function, screening for complications – foot exam, eye problems, macrovascular disease, depression)? [PROCESS]	0<br><br>Not at all	1<br><br>Some elements	2<br><br>Most elements	3<br><br>All elements but not consistently	4<br><br>All elements and most of the time	5<br><br>All elements and systematically	-Asking the health care staff	
2.7. To what extent are **medication reviews undertaken in elderly** with T2D in order to avoid polypharmacy, hypoglycemia and renal dysfunction? [PROCESS]	0<br><br>No medication reviews	1<br><br>For some patients based on HCW's own initiative	2<br><br>Routinely done	3<br><br>Routinely done and sometimes seeking advice from pharmacist	4<br><br>This is sometimes done in a multidisciplinary setting (including pharmacist)	5<br><br>This is routinely done in a multidisciplinary setting (including pharmacist)	-Asking the health care staff	
**Component 3**: **Health education and counselling to patients with T2D by non-physician care providers**	**No or little implementation**		**Moderate implementation**		**Almost complete or full implementation**			
3.1. To what extent, do patients with T2D receive **information on how to reduce health risks** by non-physicians? *(information on chronic disease management and lifestyle support)* [PROCESS]	0<br><br>Not at all	1<br><br>Only informal education is given	2<br><br>Within consultation by a non-physician	3<br><br>Structured individual education by a non-physician is scheduled for every patient	4<br><br>A multidisciplinary team provides structured education to groups of patients	5<br><br>Group sessions by a multidisciplinary team, the sessions are quality assured (reviewed) and made use of interactive techniques such as videos, discussion, etc.	-Asking nurses or community health workers or other HCWs<br>-Checking with patients<br>-Observation (if possible)	

3.2. To what extent, are patients **informed about the chronic condition** of T2D by non-physicians (including the expected course, expected complications, and effective strategies to prevent complications and manage symptoms)? *(information on the prognosis)* [PROCESS]	0<br><br>Not at all	1<br><br>Only informal education is given	2<br><br>Within consultation by a non-physician	3<br><br>Structured individual education by a non-physician is scheduled for every patient	4<br><br>A multidisciplinary team provides structured education to groups of patients	5<br><br>Group sessions by a multidisciplinary team, the sessions are quality assured (reviewed) and made use of interactive techniques such as videos, discussion, etc.	-Asking nurses or community health workers or other HCWs<br>-Checking with patients<br>-Observation (if possible)	
3.3. To what extent, are non-physicians **trained to provide health education and counselling** to patients with T2D? [STRUCTURE/EDUCATION]	0<br><br>Not at all	1<br><br>On the job training (formal education at college or university)	2<br><br>On the job training (formal education at college or university) and when the service started	3<br><br>as in 2 plus sporadic extra trainings on the topic	4<br><br>As in 2 plus systematically extra trainings on the topic	5<br><br>As in 2 plus systematically extra obligatory trainings on the topic, with innovative methods	-Asking nurses or community health workers or other HCWs	
3.4. To what extent, are **health education or counselling materials** accessible to non-physicians for T2D? [STRUCTURE]	0<br><br>Not available at all	1<br><br>Available (some materials)	2<br><br>All available but not accessible	3<br><br>Partially accessible to all necessary materials	4<br><br>Fully accessible to almost all necessary materials	5<br><br>Fully accessible to all necessary materials	-Asking nurses or community health workers or other HCWs to show it<br>-Observation at the facility	

**Component 4: Self-management support to patients and their informed caregivers with tools for adherence and monitoring** *Self-management support: supporting patients to self-manage their conditions (practice and reinforce)*	**No or little implementation**		**Moderate implementation**		**Almost complete or full implementation**			
4.1. To what extent, are patients offered **self-management training** for T2D (for example, to improve adherence to medications, proper nutrition, having self-monitoring tools at home, consistent exercise, tobacco cessation, and maintain other healthy behaviours)? [PROCESS]	0<br><br>Not at all	1<br><br>Little offer (only one element of the list) and not systematically offered to every patient	2<br><br>Limited offer (more than two elements in the list) but not systematically offered to every patient	3<br><br>Limited offer (more than two elements in the list) and systematically offered to every patient	4<br><br>Offer all the elements in the list but not systematically offered to every patient	5<br><br>Offer all the elements in the list and systematically offered to every patient	-Asking nurses or community health workers or other HCWs	
4.2. To what extent, do health care staff or community health workers **support patients' self-management** efforts **on a continuous basis for T2D?** [PROCESS]	0<br><br>Not at all	1<br><br>Only once when the care started	2<br><br>In most visits, but no use of telephone/apps	3<br><br>Once a year via telephone or email	4<br><br>Once per quarter via telephone call or email	5<br><br>On every visit and supported with commonly used apps	-Asking nurses or community health workers or other HCWs	
4.3. To what extent, are health care staff or community health workers **competent to perform self-management training?** [STRUCTURE/EDUCATION]	0<br><br>Not at all	1<br><br>Know but cannot perform (no confidence or lack of equipment or materials	2<br><br>Can perform with guidance from others	3<br><br>Can perform limited training lessons	4<br><br>Can perform almost all the training lessons	5<br><br>Can perform all the training lessons	-Asking nurses or community health workers or other HCWs	

4.4. To what extent, does the patient have **access to materials for self-monitoring for T2D**, for instance, glucose meter/ glucose test strips?<br>[STRUCTURE]	0<br><br>Not at all	1<br><br>Exist in theory but access for patients is not organised	2<br><br>Access for some patients to all materials needed but refills lack	3<br><br>Access for some patients to all materials needed including refills (strips, lancets)	4<br><br>Well-organised with access for all patients to all materials needed but refills lack	5<br><br>Well-organised with access for all patients to all materials needed including refills (strips, lancets)	-Asking nurses or community health workers or other HCWs	
4.5. To what extent are **informal caregivers/non-medical** involved in the self-management process for T2D?<br>*(i.e. family, social worker, community workers, organisations - it must be informal and not part of the health care system)*<br>[PROCESS]	**0**<br><br>Not at all	**1**<br><br>Occasionally involved but no health knowledge	**2**<br><br>Occasionally involved with limited health knowledge	**3**<br><br>Fully involved with limited health knowledge	**4**<br><br>Fully involved with full knowledge but not receiving any supporting materials	**5**<br><br>Fully involved with full knowledge and receiving supporting materials	-Asking nurses or community health workers or other HCWs	
4.6. Are the **concerns of patients and families** addressed?<br>[PROCESS]	0<br><br>Not at all	1<br><br>Is not consistently done	2<br><br>Is provided for specific patients and families through referral	3<br><br>Is provided for specific patients and families in the health facility	4<br><br>Is encouraged, and peer support, groups and mentoring program is available	5<br><br>Is an integral part of primary care and includes systematic assessment and routine involvement in peer support, group or mentoring program	-Asking nurses or community health workers or other HCWs	

4.7. Are **patient treatment plans** agreed with patients, reviewed and written down?<br>[PROCESS]	0<br><br>Patient treatment plans not expected	1<br><br>Patient treatment plans only sometimes written down	2<br><br>Patient treatment plans achieved through a standardized approach for the majority of patients	3<br><br>Patient treatment plans with clinical goals established collaboratively with patients	4<br><br>Patient treatment plans with clinical goals and self-management established collaboratively with patients	5<br><br>Patient treatment plans with clinical goals, self-management, and follow-up care established collaboratively with patients	-Asking nurses or community health workers or other HCWs	.
**Component 5**: **Structured collaboration between health care workers, community actors, and patients and caregivers**	**No or little implementation**		**Moderate implementation**		**Almost complete or full implementation**			
5.1. To what extent, is there an identified **"care coordinator"** who serves as the overseer and director of a patient's care, ensuring that efforts of all involved health care workers, community actors, and patients and caregivers are integrated and coordinated for T2D?<br>[STRUCTURE]	0<br><br>Not exist	1<br><br>Exist but not active	2<br><br>Exist and active only when triggered	3<br><br>Exist and active occasionally	4<br><br>Exist and active but not structured	5<br><br>Exist and active and structured	-Asking the health care staff<br>-Checking in the community	
5.2. To what extent, do the **health care organization and the community** have **complementary functions**, that is, community organizations fill gaps in services that are not provided in formal health care for T2D?<br>[PROCESS]	0<br><br>Not at all	1<br><br>Community effort exists but not relevant to the gap	2<br><br>Community effort exists and relevant but unable to fill the gap	3<br><br>Community effort exists and able to limitedly fill the gap	4<br><br>Community effort exists and able to almost fill the gap	5<br><br>Community effort exists and able to completely fill the gap	-Asking the health care staff and patients<br>-Checking in the community	

5.3. To what extent, are **referral practices** systematically organized for T2D?<br>[PROCESS]	0<br><br>Not at all	1<br><br>Oral referral only	2<br><br>Written referral and only one direction	3<br><br>Written referral for two directions (without tele-communication)	4<br><br>Written referral organised for two directions (confirmed by tele-communication) and case by case	5<br><br>Written referral organised systematically for two directions for all cases (confirmed by tele-communication)	-Asking the health care staff	
5.4. To what extent does **cooperation between health care workers and other professionals and community actors** occur for T2D?<br>[PROCESS]	0<br><br>No cooperation	1<br><br>Little cooperation without regular discussion	2<br><br>Moderate cooperation within the team with regular discussion	3<br><br>Full cooperation within the team but not across	4<br><br>Cooperation within and across the team	5<br><br>Multi-disciplinary cooperation within the team and across all levels	-Asking the health care staff	
5.5. To what extent is the traditional **hierarchy flattened** and moved away from physician dominated models for T2D?<br>[STRUCTURE]	0<br><br>Specialists are dominating and in the first line	1<br><br>Specialists are central for some patients, and for some general practitioners play the central role	2<br><br>General practitioners are central, and other HCWs play a minor role	3<br><br>General practitioners are central, and other HCWs play a big role	4<br><br>There is a multidisciplinary team and everyone is considered equal	5<br><br>HCWs with special training in chronic care take the lead	-Asking the health care staff	
**Component 6: Organisation of care, delivery system design and clinical information systems**	**No or little implementation**		**Moderate implementation**		**Almost complete or full implementation**			

6.1. To what extent are ongoing **quality improvement** routine activities among health care workers organised?<br>[PROCESS]	0<br><br>No quality improvement	1<br><br>New rules to improve care quality are sometimes set from management	2<br><br>When a problem pops up a quality improvement activity is sometimes undertaken	3<br><br>When a problem pops up a quality improvement activity is often undertaken	4<br><br>Is a routine process but results from previous rounds are often not taken into account	5<br><br>Is a routine process and results from previous rounds are taken into account	-Asking the health care staff	
6.2. To what extent do **information systems** gather and organise data about epidemiology, treatment, and health care outcomes?<br>[STRUCTURE]	0<br><br>There is no registry	1<br><br>There is a registry but is not used for treatment purpose	2<br><br>The registry includes name, diagnosis, contact information and date of last contact	3<br><br>The registry includes name, diagnosis, contact information, date of last contact, treatment and outcomes	4<br><br>The registry includes all the information in 3 and also allows queries to sort subpopulations by clinical priorities	5<br><br>As in 4, the registry is also tied to guidelines which provide prompts and reminders about services needed	-Asking the health care staff	
6.3. To what extent do information systems serve a **reminder function** for patient specific prevention and follow-up services (e.g. to identify patients' needs, to follow-up and plan care, to monitor responses to treatment, and to assess health outcomes)?<br>[STRUCTURE]	0<br><br>No Information system	1<br><br>There is a system but no reminder function	2<br><br>There is a system and reminders that include general notification of the existence of a chronic illness, but does not describe services needed at time of encounter	3<br><br>There is a system and reminders that describe services needed at time of encounter, based on general guideline, not patient-specific	4<br><br>There is a system that includes specific information for each patient at the time of individual patient encounter	5<br><br>As in 4, the system also includes specific information for the health care team about adherence to patient care plan at the time of individual patient encounter	-Asking the health care staff	

6.4. To what extent is **feedback about the performance provided to the team and its members**? [PROCESS]	0<br><br>Not available	1<br><br>Non-specific to the team	2<br><br>Infrequent intervals and not delivered to the team (they need to search for the information)	3<br><br>Frequent intervals but not specific for the team and impersonally delivered (just common reports)	4<br><br>Occurs at frequent enough intervals to monitor performance and is specific to the team	5<br><br>Timely and specific to the team, routinely and personally delivered by a respected opinion leader	-Asking the health care staff	
6.5. To what extent is an **appointment system** with planned visits used? [STRUCTURE]	0<br><br>No appointment system	1<br><br>Used to schedule acute care visits, follow-up and preventive visits	2<br><br>Appointment system assures scheduled follow-up with chronically ill patients, but some patients escape the system	3<br><br>Appointment system assures scheduled follow-up with all chronically ill patients	4<br><br>Appointment system is flexible and can accommodate innovations such as customized visit length or group visits	5<br><br>Appointment system includes organisation of care that facilitates the patient seeing multiple providers in a single visit	-Asking the health care staff	
